# In Vitro Mechanical Study of Three-Dimensional Printed Invisible Dental Aligners for Crowded Dentition Problems: A Patient-Specific Study

**DOI:** 10.3390/biomimetics11020108

**Published:** 2026-02-03

**Authors:** Zelafy Reynosa, Hong-Seng Gan, Muhammad Hanif Ramlee

**Affiliations:** 1 Bone Biomechanics Laboratory (BBL), Department of Biomedical Engineering and Health Sciences, Faculty of Electrical Engineering, Universiti Teknologi Malaysia, Johor Bahru 81310, Malaysia; reynosa@graduate.utm.my; 2 School of AI and Advanced Computing, Xi’an Jiaotong—Liverpool University, Suzhou 215000, China; hongseng.gan@xjtlu.edu.cn; 3 Rehabilitation Nexus (RehabNex) Research Group, Universiti Teknologi Malaysia, Johor Bahru 81310, Malaysia

**Keywords:** orthodontics, orthodontic appliances, clear aligners, malocclusion, dental materials, mechanical properties, additive manufacturing

## Abstract

Clear aligners are a popular alternative to fixed orthodontic appliances; however, technical data on the optimal final aligner shell thickness for directly printed aligners remain limited. This in vitro experimental pilot study evaluated the mechanical response of patient-specific, directly 3D-printed aligners of four nominal shell thicknesses (0.04, 0.06, 0.08, and 0.10 mm) fabricated from BioMed Clear resin. A single subject with dental crowding was scanned and a set of aligner shells was designed and printed (*n* = 3 per thickness). Compressive tests up to 1000 N were performed and compressive extension (mm) recorded; group means ± SD were compared by means of one-way ANOVA. No statistically significant differences in compressive extension were found among the four thickness groups (ANOVA, F(3,8) = 2.242, *p* = 0.161). The 0.08 mm group showed a lower mean compressive extension in this dataset, but the difference did not reach statistical significance; given the small sample size and single-subject nature of the study, this result should be considered exploratory. This recent study clarifies printing and post-processing parameters and highlights limitations and directions for future work.

## 1. Introduction

Orthodontic treatment has increasingly shifted toward aesthetic and patient-centered approaches, with clear aligners becoming a common alternative to fixed appliances. Compared with conventional bracket-based systems, clear aligners are associated with improved aesthetics, better patient acceptance, and easier maintenance of oral hygiene. These advantages have contributed to their widespread use, particularly among adult patients [1].

Despite their clinical effectiveness, fixed orthodontic appliances continue to present challenges related to oral hygiene, as brackets and archwires facilitate plaque retention and complicate cleaning [2]. This buildup can cause white spot lesions, cavities, and significant gum damage, especially around the upper lateral incisors and canines. In the last several decades, clear aligner therapy has been created to meet the cosmetic and comfort needs of adult orthodontic patients. This therapy—based on a unique set of clear, thin, and removable aligners manufactured using plastic—is designed to shift the patient’s teeth into the appropriate position [3]. These dental aligners are changed every two weeks and worn for about 20 h a day. The teeth will move 0.25 mm to 0.3 mm with each aligner [4]. Aligner therapy may treat minor non-extraction situations more quickly and effectively [5].

Recent advances in digital dentistry have introduced additive manufacturing as an alternative to conventional thermoforming workflows. While three-dimensional (3D) printing is well established for producing dental models and aligner molds, direct fabrication of clear aligners remains comparatively underexplored [6]. One of the important parameters to consider when printing clear aligners is the layer height of a 3D-printed dental aligner. It influences the final product’s accuracy and strength. Although there are other elements influencing the accuracy of 3D-printed dental models, the layer thickness setting may be one of the most important under the assumption that each printer has the same XY resolution and printing environment [7]. Existing studies investigating 3D-printed aligners are limited in number and primarily focus on general material properties or simplified test specimens rather than clinically relevant aligner geometries. Moreover, most available investigations evaluate a narrow range of printing parameters, making it difficult to draw conclusions about how manufacturing settings influence mechanical behavior [6,7].

Among the parameters involved in 3D printing, layer height is considered a key factor affecting interlayer bonding, surface morphology, and mechanical performance [4,5]. Although a small number of studies have assessed the mechanical properties of 3D-printed orthodontic materials across selected thickness or layer settings, these investigations typically examine standardized samples rather than patient-specific aligners. As a result, there is limited evidence on how layer height influences the mechanical strength of directly printed aligners shaped to real dental anatomies, which are subjected to complex stresses during clinical use [7,8].

In addition, BioMed Clear resin, a biocompatible photopolymer increasingly used in medical applications, has not been sufficiently investigated for direct fabrication of orthodontic aligners. While its general material characteristics are documented, its mechanical performance when printed into patient-specific aligner geometries—and how this performance varies with layer height—remains unclear.

Therefore, the aim of the present study is to evaluate the mechanical strength of directly 3D-printed clear aligners fabricated from BioMed Clear resin at selected layer heights using patient-specific geometries. By focusing on a controlled experimental design and clinically relevant aligner forms, this study seeks to provide preliminary mechanical data that may inform future optimization of printing parameters and guide further experimental and clinical investigations.

## 2. Materials and Methods

### 2.1. Subject Recruitment and Dentition Scanning

**Figure 1:**
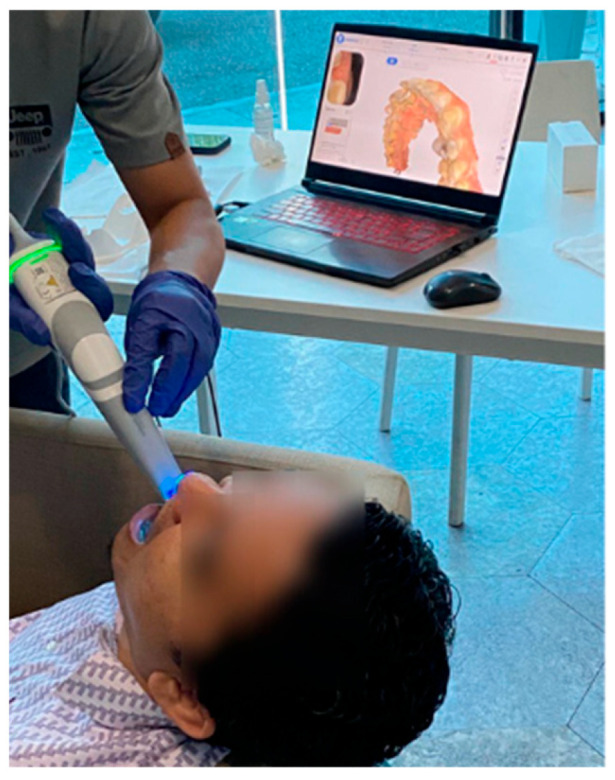
Three-dimensional scanning of the subject’s dentition by using a Shining 3D scanner.

One subject (male, 35 years old, severity index = 4, and never used braces before) meeting the inclusion criteria was recruited for dentition scanning. The subject was classified as having crowding teeth, a common specific malocclusion in which a lack of space causes teeth to overlap or turn. Informed consent was obtained from the participant, and ethical approval (approval no.: UTMREC-2025-150, approval date: 12 August 2025) was obtained early, in compliance with the ethical guidelines outlined in the Declaration of Helsinki. The scanning process was conducted using a 3D dental intraoral scanner (Model: Aoralscan 3, Shining 3D, China) [9]. The subject was seated comfortably (Figure 1), ensuring minimal head movement, and the dental scanner was connected to a laptop with dedicated software. Both maxillary and mandibular arches were scanned, and the resulting STL files were aligned using the standard alignment procedure provided by CAD software (Mimics 10, Materialise, Belgium). The occlusal relationship captured during scanning was preserved during alignment and was not modified. Only the maxillary arch was used for subsequent analysis, in accordance with the study objectives. The scanning resolution was set as a default setting from the 3D scanner (scan depth: ~22 mm range, and scan resolution ≤ 0.01 mm). The scanning process was guided by the scanner’s software, which allowed for real-time visualization of the scanned areas. The resulting 3D scans were exported in stereolithography (STL) format.

### 2.2. Aligner Modelling

**Figure 2:**
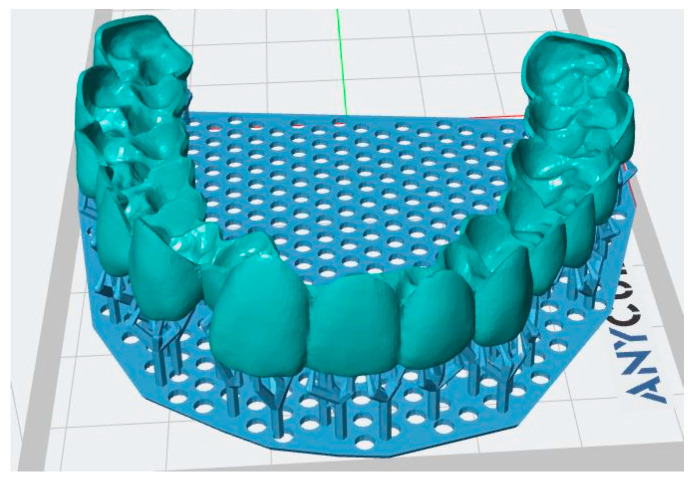
The 3D design of the clear aligners with it supports from the subject’s dentition.

The scanned STL files of the teeth were employed to create dental aligners through computer-aided design (CAD) software (3-matic, Materialise, Belgium) [10]. Both the maxillary and mandibular dental arches were scanned, and the resulting digital models were aligned using the software’s standard alignment procedure. Although the occlusal relationship between the upper and lower arches was preserved during the scanning and alignment process, only the maxillary arch was utilized for further analysis in this study. No simulated tooth movement was incorporated during CAD modelling, and the aligners were generated as passive shells that replicated the original dental anatomy, without any programmed displacement or orthodontic force application. The CAD workflow involved loading the STL file, indicating any missing teeth, assessing arch length, and defining the boundaries of the teeth for accurate aligner design [11]. The digital models were further refined in 3-Matic software by removing unnecessary elements, closing surface gaps, and smoothing the overall geometry. In the present study, aligner thickness refers to the uniform thickness of the aligner shell and not to the printer’s layer height parameter. Uniform thickness was ensured by applying a constant shell thickness parameter across the entire tooth surface, including areas of complex geometry. The aligner thickness was incrementally varied to produce four thicknesses (0.04 mm, 0.06 mm, 0.08 mm, and 0.10 mm). The final STL files were imported into Anycubic Photon Workshop software for 3D printing, and support structures were added, as shown in Figure 2.

### 2.3. Three-Dimensional Printing and Post-Processing

**Figure 3:**
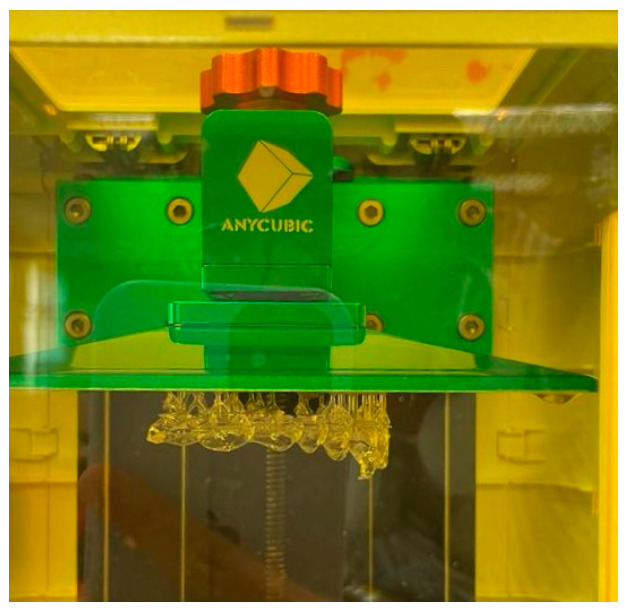
The 3D-printed clear aligner using the SLA printer.

**Figure 4:**
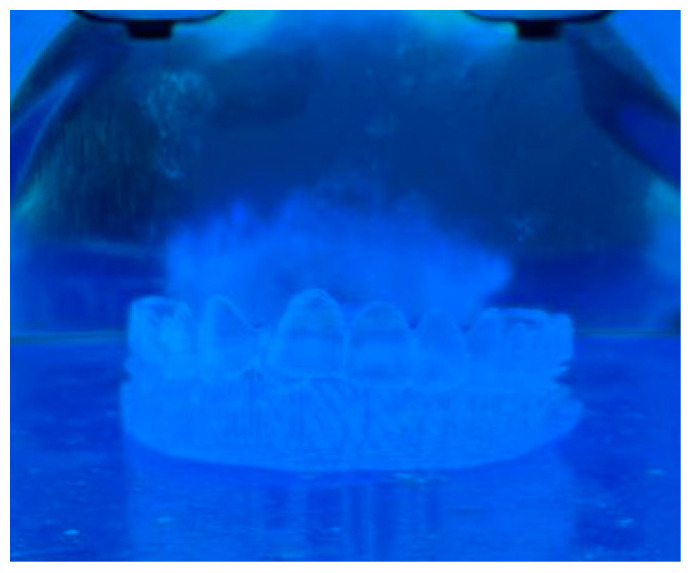
The printed aligner was exposed to UV light for 20 min.

The aligners underwent 3D printing using an Anycubic Photon Workshop stereolithography (SLA) printer, employing BioMed Clear resin (FormLabs, Somerville, MA, USA). Figure 3 and Figure 4 show the printed aligners and post-processing steps. Each layer thickness had three samples printed for more accurate results when undergoing compression testing. To ensure precise printing, support structures were incorporated into the models using the software [12]. All aligners were printed using the same default build orientation and support generation settings provided by the software; however, a detailed optimization or comparison of build orientation and support placement strategy was not performed. All experimental groups were printed under identical printing conditions and build settings to ensure consistency. After printing, the aligner models were cleansed with 99.9% isopropyl alcohol (IPA) for 10 min to eliminate any residual resin and then left to air-dry. Subsequently, a post-curing process took place in a UV-light chamber at 30 °C for 20 min to optimize material polymerization and biocompatibility as shown in Figure 4 [13]. Finally, the aligners’ support structures were removed using standard cutting tools [8,14]. No additional dimensional accuracy or quality control measurements, such as post-print thickness verification after curing, were conducted; this limitation has been acknowledged in the study.

### 2.4. Mechanical Testing

**Figure 5:**
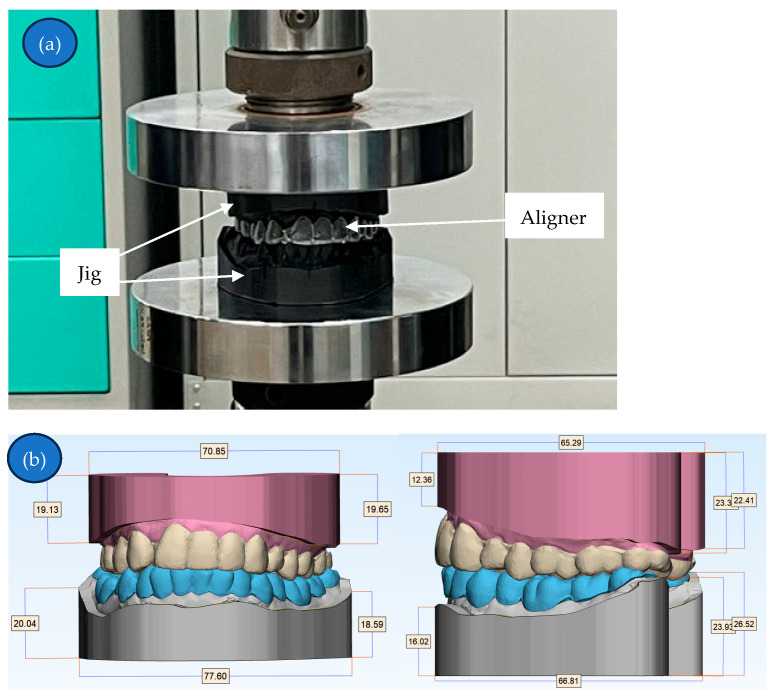
(**a**) The compressive testing equipment set up, and (**b**) the dimension (in mm) of the jig.

The mechanical strength of the aligners underwent evaluation using an Instron universal testing machine. Maximum voluntary bite force in adults has been reported at approximately 738 N in the molar region, confirming that the 1000 N compression load applied to each aligner thickness in this study exceeds average human biting forces [12]. A jig, crafted in 3-Matic and fabricated via 3D printing, firmly secured the aligner specimens between two parallel flat plates of the Instron machine. Compression testing was executed at a rate of 2 mm/min, with the measurement of compressive load (N) and compressive extension (mm) for subsequent analysis [13]. The compression testing setup and jig dimension (in mm) is illustrated in Figure 5a and Figure 5b, respectively. Data analysis was conducted utilizing one-way ANOVA.

## 3. Results

### 3.1. Compressive Testing

The aligners were tested at various layer thicknesses, including 0.04 mm, 0.06 mm, 0.08 mm, and 0.10 mm. Each aligner was subjected to a consistent compressive load of 1000 N. Data gathered during the compression tests included the compressive load (N) and the corresponding compressive extension (mm).

#### 3.1.1. Statistical Analysis

The gathered data underwent statistical analysis using one-way Analysis of Variance (ANOVA) to identify disparities among the tested aligner layer thicknesses. This analysis aimed to establish whether significant differences existed in the means across groups. ANOVA was employed to evaluate variations and distinctions among the means of each layer thickness group. The interpretation of *p*-values from this analysis determined no significant differences between groups, as detailed in Table 1.

#### 3.1.2. Results and Interpretation

The ANOVA analysis revealed a *p*-value exceeding 0.05, suggesting insufficient evidence to reject the null hypothesis regarding the subject’s crowded teeth. This indicates that no statistically significant differences were observed in mean compressive extension across the evaluated aligner thicknesses. Table 2 summarizes the mean and standard deviation of compressive extension for each thickness group. Among the four evaluated thicknesses, the 0.08 mm aligner exhibited a lower mean compressive extension, while greater variability was observed in the 0.06 mm and 0.01 mm samples; however, these differences were not statistically significant. The observed inconsistencies in mean values may be associated with variations in jig positioning under load, potentially arising from irregularities in tooth alignment, which may have contributed to misalignment and increased measurement variability. Additional variability may also be related to printing conditions. In this investigation, the longest printing duration was approximately 2 h for a layer thickness of 0.10 mm. The minimum exposure time required for successful printing was 25 s, as shorter exposure times resulted in failed prints consisting only of support structures. Prolonged printing durations in SLA fabrication may predispose the resin to over-curing, which can increase brittleness and reduce material flexibility, thereby influencing mechanical performance.

**Figure 6:**
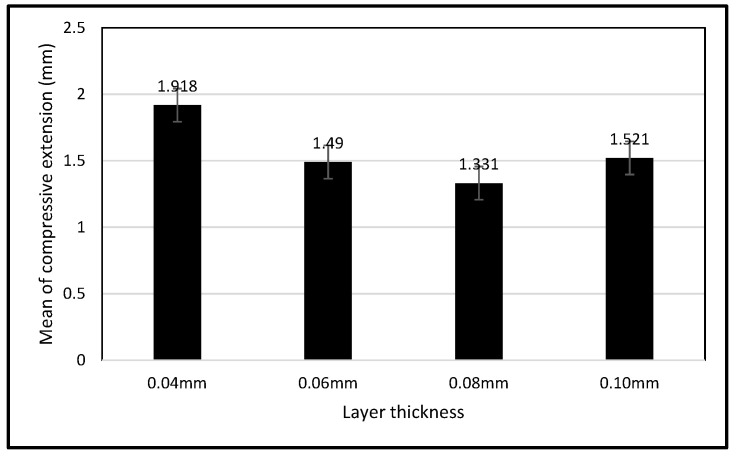
The bar chart of mean compressive extension (mm) over thickness (mm) for the subject.

**Figure 7:**
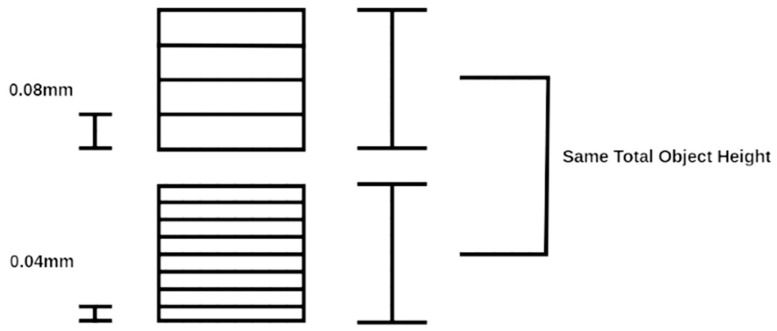
Visualization of the increase in layer number as resolution is decreased.

The mean compressive extension values for each aligner thickness are illustrated in Figure 6. Although the 0.08 mm group exhibited a lower mean compressive extension compared with the other thicknesses, this difference was not statistically significant (one-way ANOVA, *p* > 0.05). The 0.04 mm aligner exhibited the highest mean compressive extension among the tested groups. When higher-resolution settings are selected in the z-direction, the number of layers required to fabricate a given geometry increases. The observed variation between the 0.04 mm and 0.08 mm thicknesses may be related to differences in layer thickness during the additive manufacturing process, whereby thinner layers require a higher number of printed layers to achieve the same geometry. For example, reducing the layer height from 0.08 mm to 0.04 mm approximately doubles the number of printed layers (Figure 7). An increased number of layers may elevate the likelihood of printing errors, artifacts, or cumulative inaccuracies during fabrication, which could ultimately reduce the dimensional accuracy of the printed aligner [15]. Future studies should further investigate location-specific deviations and isolate printing-related sources of variability.

Regarding variations in layer thickness, the 0.08 mm thickness consistently displayed low compressive extension, indicating promising mechanical strength. Consequently, the 0.08 mm aligner thickness was deemed appropriate based on this assessment.

## 4. Discussion

The assessment of mechanical characteristics in dental aligners across varying layer thicknesses, ranging from 0.02 mm to 0.1 mm, produced results contrary to initial predictions. The expected pattern of decreased compressive extension as aligner thickness increased, suggesting improved mechanical resilience, was not observed in this investigation. Numerous constraints in the experimental arrangement and materials were recognized, which potentially influenced these unanticipated findings.

Compression testing is widely regarded as a critical method for evaluating the mechanical behavior of dental aligners, as it provides insight into their strength, durability, and functional performance. Previous research has demonstrated that the mechanical properties of aligners can vary depending on the 3D printing technology employed. For instance, Zinelis et al. [16] reported notable differences in aligner mechanical performance when fabricated using five different 3D printers, with the Slash 2 printer yielding the highest elastic modulus (2696.3 MPa). In contrast, the present study employed a single stereolithography (SLA) system (Anycubic Photon Workshop), which restricts direct comparison with studies utilizing multiple printing platforms. Their results indicated a minimum tooth displacement of 0.2 mm across aligner thicknesses ranging from 0.3 to 0.6 mm. However, in the present study, the lowest compression extension measured was 1.331 mm for 0.08 thickness. This demonstrates that lower thicknesses of the aligner contribute to high extension.

### 4.1. Experimental Limitations and Interpretations

#### 4.1.1. Jig Positioning

A notable constraint was the lack of consistency in the positioning of the jig on the lower plate during compression testing. The irregular shape of the teeth on the jig required a consistent jig position for each test to distribute force evenly across the aligner. This inconsistency probably led to uneven force distribution, which influenced the varying measurements observed across different thicknesses. Marking the plate to establish fixed jig positions could potentially resolve this problem by ensuring uniform force distribution and enhancing the reliability of the measurements.

#### 4.1.2. Sample Size

In the present study, there was only one subject involved, which is one of the limitations of the study. Increasing the number of samples for each aligner thickness and subject would enable more rigorous statistical analysis, including the calculation of representative mean values for each thickness group. A larger sample size would also facilitate the identification of experimental variability and reduce uncertainty in the measurements, thereby improving result accuracy [17]. Furthermore, an expanded dataset would enhance the generalizability of the findings and provide stronger evidence regarding the mechanical properties. Consequently, future studies should incorporate larger sample sizes to improve the reliability and precision of their conclusions. However, in this study, we reported a pre-clinical result that may lead to future investigations by other researchers to further the research.

#### 4.1.3. Material Limitations

Limitations also encompassed material selection, as Dental LT Clear resin was suggested but inaccessible regionally due to cost and availability constraints. Substituting BioMed Clear resin introduced notable inconsistency due to differences in mechanical properties [17]. This deviation from the intended material hindered result comparison with prior studies. However, previous research has reported that BioMed Clear resin possesses favorable characteristics for a range of medical 3D printing applications, indicating its capability to produce durable and high-quality medical devices [18,19,20]. However, its application in the fabrication of dental aligners remains largely unexplored. The present study is among the early efforts to evaluate the feasibility of BioMed Clear resin for dental aligner production and demonstrate encouraging outcomes with respect to mechanical performance and cleaning effectiveness. These results indicate that BioMed Clear resin may represent a promising material candidate for future dental applications. Nevertheless, further investigations are required to comprehensively assess its suitability, particularly in terms of long-term mechanical behavior, color stability, and clinical performance when used in dental aligners. Such studies would contribute to the existing literature and support the development of improved aligner materials, potentially facilitating wider adoption of BioMed Clear resin in clinical dental practice.

#### 4.1.4. Mechanical Measures

One limitation of this study concerns the outcome measures. At present, the analysis focuses solely on compression–extension (mm). Although other mechanical parameters—such as stress, strain, elastic modulus, yield point, or fracture behavior—were not included, compression–extension was deliberately selected as the primary variable, as it directly reflects the functional deformation response of the material under clinically relevant loading conditions and has been identified as the most relevant outcome in comparable studies reported in the literature [16,17]. Furthermore, compression–extension measurements provide a straightforward and reliable indicator of mechanical performance when evaluating patient-specific geometries, where complex stress distributions may be difficult to generalize. Nevertheless, it is acknowledged that limiting the analysis to a single mechanical parameter may not fully capture the complete mechanical behavior of the material. Therefore, future work should incorporate additional mechanical properties and advanced mechanical analyses to enable a more comprehensive characterization, improve inter-study comparability, and provide deeper insights into the material’s structural and functional behavior under orthodontic loading conditions.

### 4.2. Recommendations

These research limitations highlight the significance of material consistency, sample size, and experimental setup. For future research, employing Dental LT Clear resin is advised to ensure more precise outcomes and enable meaningful comparisons with the existing literature. Choosing repackaged containers over cartridges when acquiring this material could alleviate cost concerns.

Ensuring uniform force distribution and consistent results necessitates addressing jig positioning issues. Introducing marking strategies on the plate could enhance reliability and reproducibility. Augmenting sample size for each thickness, particularly per subject, would refine result precision, aiding in error identification and trend recognition.

To surmount material-related constraints, exploring alternative materials with enhanced color stability and mechanical properties is warranted. Additionally, integrating finite element analysis (FEA) alongside compression testing could offer a comprehensive assessment, validating experimental findings [21]. A comparative evaluation between simulation and experimental data may yield more precise mechanical property assessments.

In conclusion, this study illuminates the complexities of evaluating dental aligner mechanical properties. The unexpected outcomes underscore the importance of meticulous experimental design, material uniformity, and comprehensive sample sizes. By addressing these constraints and integrating advanced analytical techniques, future research endeavors can strive for more accurate and dependable evaluations of dental aligner properties.

## 5. Conclusions

This study investigated the compressive mechanical behavior of clear aligners fabricated with different layer thicknesses using BioMed Clear resin. The statistical analysis revealed no significant differences in compressive extension among the evaluated thickness groups (*p* > 0.05). While minor descriptive variations in mean values were observed, these trends were not statistically supported and therefore do not justify the identification of an optimal thickness. The results further highlight the influence of experimental factors, including jig positioning, printing conditions, and limited sample size within a single-subject design. Consequently, the findings should be regarded as preliminary and serve primarily to inform future studies employing larger sample sizes and improved experimental control.

## Figures and Tables

**Table 1:** Overall one-way ANOVA results.

	DF	Sum of Squares	Mean Square	F Value	Prob > F
**Between Groups**	3	0.562	0.187	2.242	>0.05
**Error**	8	0.668	0.083	-	-
**Total**	11	1.23	-	-	-

**Table 2:** Descriptive statistics of compressive extension for each aligner layer thickness.

Layer Thickness (mm)	n	Mean Compressive Extension (mm)	SD (mm)
0.04	3	1.918	0.12
0.06	3	1.490	0.11
0.08	3	1.331	0.10
0.10	3	1.521	0.13

## Data Availability

The data presented in this study are available upon request from the corresponding author.
